# Social support and age influence distress outcomes differentially across urban, regional and remote Australia: an exploratory study

**DOI:** 10.1186/1471-2458-12-928

**Published:** 2012-10-30

**Authors:** Joanne Allen, Kerry J Inder, Terry J Lewin, John Attia, Brian J Kelly

**Affiliations:** 1 Centre for Translational Neuroscience and Mental Health, University of Newcastle and Hunter New England Health, Newcastle, NSW, Australia; 2 Hunter Medical Research Institute, Newcastle, Australia; 3 Centre for Clinical Epidemiology and Biostatistics, University of Newcastle and Hunter New England Health, Newcastle, NSW, Australia

**Keywords:** Rural health, Psychological distress, Social support, Aged 55 and over

## Abstract

**Background:**

The variation of determinants of mental health with remoteness has rarely been directly examined. The current research aims to examine whether the association of psychosocial factors with psychological distress outcomes varies with increasing remoteness.

**Methods:**

Participants were persons aged 55 and over from two community cohorts sampling from across rural and urban New South Wales (N = 4219; mean age = 69.00 years; 46.1% male). Measures of social support from these studies were calibrated to facilitate comparison across the sample. Remoteness was assessed using a continuous measure, the Accessibility/Remoteness Index of Australia. The association between demographic characteristics, social support, remoteness, and their interactions with remoteness in the prediction of high psychological distress (cut-off > 21 on the Kessler 10) were examined using logistic regression.

**Results:**

Not being in a married or defacto relationship (OR 0.69; 99% CI 0.51-0.94), lower education (OR 0.52; 99% CI 0.38-0.71) and decreased social support (OR 0.36; 99% CI 0.31-0.42) significantly predicted psychological distress. There was a significant interaction of age and remoteness (OR 0.84; 99% CI 0.67-1.00), indicating that as remoteness increases, older persons are less likely to be highly distressed, as well as a significant interaction of social support and remoteness (OR 1.22; 99% CI 1.04-1.44), indicating that as remoteness decreases, persons with low levels of social support are more likely to be highly distressed.

**Conclusions:**

Remoteness may moderate the influence of social support and age on psychological distress outcomes.

## Background

Rural settings have been characterised as having distinct social, environmental and cultural features which may have a significant impact on the wellbeing of persons living in these regions. Rural populations report high levels of social capital which may be protective against poor mental health outcomes [1-3]. However, qualitative evidence from Australia suggests rural populations possess a culture of self-reliance and stoicism which may exacerbate social isolation and impede help seeking behaviours [4,5]. In addition to facing substantial geographical barriers to accessing health and mental health services and decreased opportunities for social interaction, rural populations are also at increased risk of occupational injury and stress due to adverse environmental conditions [6]. Further, decreased opportunities in rural areas have led to increased migration of younger generations away from rural communities [7], resulting in increasingly older age profiles in these areas [8]. How these characteristics of remote communities interact to influence psychological distress is not clear.

There is little evidence of an influence of remoteness on psychological distress [9-11]. Recent reviews of the evidence have suggested variously that rates of mental illnesses are higher in urban areas compared to rural areas [12], that there is little evidence of an urban-rural differential in prevalence of mental health disorders [13], and that suicide rates for men are higher in rural compared to urban areas but do not differ for women [13]. Such variations may be attributable to methodological differences between studies, including differing classifications of what is ‘urban’ and what is ‘rural’, as well as variations in the environmental and cultural conditions between countries. While it is unclear whether there is an urban-rural difference in the incidence of mental illness in Australia, there is growing evidence that the influence of individual level demographic and social characteristics on psychological wellbeing may be moderated or ‘exacerbated’ by the social and physical environment [10,13,14]. Recent data from the Australian Rural Mental Health Study [9] indicates that individual demographics, recent adverse events and social capital account for a substantial proportion of variability in wellbeing among a non-metropolitan Australian sample. While such research highlights potential targets for influencing positive mental health outcomes in rural environments, few studies to date have attempted to assess how remoteness may influence the effects of known individual level determinants of health.

Several studies have observed that the association of demographic characteristics, such as gender [2,15-21], marital status [17] and social class [17] with mental health outcomes vary between urban and rural environments. Indeed three-way interactions of remoteness, gender, ethnicity, as well as remoteness, gender and household composition in determining depression symptomology have been observed in a national survey of American households [22]. Studies examining the influence of individual level social factors on depression by remoteness demonstrate a negative association between depression and social support in both urban and rural environments [3,18,21,23,24]. A South Korean cohort observed social support to be strongly associated with depression in those with lifetime rural residence, but not lifetime urban residence [18]. Such studies suggest while social support is an important determinant of wellbeing, the strength of its protective effect may depend on the social and physical environment in which it is experienced and may be more important for those in rural areas. To determine whether these observations highlight important targets for intervention in Australia, the association between social support and mental health outcomes need to be explored in an Australia sample representative of the spectrum of urban-remote communities.

The current study examined whether individual level characteristics such as demographics and ratings of social support influence psychological distress outcomes differentially across urban-remote regions of Australia in a sample of older persons. Data from the Australian Rural Mental Health Study (ARMHS) [25] and comparable data from a study of urban-inner regional areas of Newcastle, NSW, known as the Hunter Community Study (HCS) [26] were combined into a single harmonized dataset. Initially, psychosocial measures that were common to these studies are described. Conceptually related baseline measures were calibrated to obtain a common measure of that construct, guided by data from a common follow-up phase conducted by these studies. How these psychosocial characteristics relate to indices of psychological wellbeing, and how these associations may vary with remoteness was examined. It was hypothesised that there would be an interaction of individual level characteristics such as demographic indices and individuals’ ratings of social support with indices of community remoteness in the prediction of high psychological distress. These findings inform us of risk factors that may be important foci for intervention across urban-remote regions of Australia.

## Methods

### Sample

Self-report postal survey data from two population-based cohort studies conducted in New South Wales, Australia were combined to undertake the current study: the Hunter Community study (HCS) [26]; and the Australian Rural Mental Health Study (ARMHS) [25]. Detailed descriptions of sampling, recruitment, and methods employed by these studies can be obtained from their baseline descriptive papers [25,26]. Briefly, the HCS is a study of persons aged 55-85 years residing in Newcastle, New South Wales, and was designed to assess a range of biopsychosocial aspects of aging. The ARMHS is an investigation of persons aged 18 years and older residing in non-metropolitan New South Wales and was designed to assess mental health and wellbeing in rural and remote regions by over-sampling from remote and very remote populations. Both the HCS and ARMHS randomly selected potential participants from the New South Wales state electoral roll. Introduction and recruitment letters were sent to individuals by post and non-responding individuals were followed-up with telephone calls. Informed written consent was obtained from all participants. Overall response rates of 44.5% (N = 3253) and 27.3% (N = 2639) for the baseline HCS and ARMHS samples respectively were achieved, with both samples having comparable rates of uncontactable or excluded persons (HCS 26.9% and ARMHS 25.2%). Within the ARMHS sample, among those who were contactable and met study inclusion criteria, participation rates varied by age group (under 55 years: 25.4%; 55-70 years: 32.4%; over 70 years: 20.1%). A comparable pattern emerged within the HCS sample, with responders tending to be slightly younger than non-responders (66.3 vs. 68.6 years) [26]. To maintain comparability with the HCS and address the aims of the current research, only participants aged 55 years and over from the ARMHS cohort (54.3%) were considered for inclusion in the current analysis.

Following ethical approval (University of Newcastle Human Research Ethics Committee, and Hunter New England Area Health Human Research Ethics Committee), baseline survey data from the HCS and ARMHS were combined. For the purposes of the current study, only those participants who provided complete information on key model variables age, gender, social support scale data and psychological distress were included in analyses. Our study is therefore based on a population of N = 4219 adults (HCS N = 3033; ARMHS N = 1186). Within this dataset, preliminary comparisons revealed that the cohorts did not differ in age (F(1, 4218) = .905, p = .341), gender (*X*^2^(1) = 3.56, p = .06), or the proportion of persons in a married or defacto relationship (*X*^2^(1) = .867, p = .874). However, a greater proportion of participants in the HCS had completed high school or higher education compared to the ARMHS (77.7 vs. 61.4%; *X*^2^(1) = 109.45, p < .001), an observation that is consistent with the lower rates of Australian high school completion with increasing remoteness [27].

### Measures

#### Primary outcome variable: Psychological distress

Psychological distress was assessed using the Kessler 10 (K10) [28] in both cohorts. The K10 is a 10-item self-report questionnaire that assesses the frequency of psychological distress over the past four weeks using a 5-point Likert scale. Scores range from 10 to 50, with higher scores denoting greater psychological distress. The K10 has been used extensively as part of the World Health Organization World Mental Health surveys [29], has been shown to be sensitive to non-specific psychiatric distress [28,30], and normative data for Australian populations have been developed [30,31]. A cut-off score of > 21 was used to indicate high psychological distress in the current study. Data from the 2007 National Survey of Mental Health and Wellbeing suggests that this cut-off is associated with a 63% likelihood of meeting 12 month ICD-10 criteria for any affective, anxiety or substance use disorder in an Australian community sample (compared to 15% of persons scoring < 21), with 9.5% of respondents rated in this category [31]. Data from the 2003 New South Wales Population Health Survey found 8.3% of participants aged 50 years and over scored above this cut-off [32].

#### Independent variables

##### Demographic characteristics

Self-reported demographic information, including age, gender, education and marital status, were assessed in both cohorts.

##### Social support

The HCS and ARMHS collected conceptually related baseline social support measures assessing participant’s network and personal support relationships. Network support was assessed using the Berkman Social Network Index [33] in the ARMHS cohort and using the Network sub-scale of the abbreviated Duke Social Support Index [34] in the HCS cohort. These scales are comprised of similar items assessing the number of friends and relatives who may be available to the individual to provide social support, the frequency of contact with these individuals, and participant’s involvement in organised social groups. Personal support relationships were assessed using the Availability of Attachment subscale of the Interview Schedule for Social Support [35] in the ARMHS cohort and using the Satisfaction subscale of the abbreviated Duke Social Support Index [34] in the HCS cohort. Both the HCS and ARMHS assessments of personal support assess participant’s access to close personal relationships with persons who could provide emotional support. The HCS assessment additionally assessed participant’s feelings of belonging and involvement with friends and family.

Under the auspices of the Extending Treatments, Education and Networks in Depression project (xTEND) [36], a common three year follow-up phase was conducted, which included administration of a range of baseline measures from both cohorts. Preliminary follow-up data from the HCS, representing the first N = 2031 surveys returned at three year follow-up, was used to calibrate baseline indices of social support so that the influence of social support on psychological distress could be assessed across studies at baseline. These preliminary analyses are reported at the beginning of the results.

##### Remoteness

The Accessibility/Remoteness Index of Australia (ARIA+) [37] was used to provide a postal-area level index of participant remoteness for both cohorts. The ARIA+ is a continuous index score ranging from 0-15 (higher scores indicating greater remoteness) that is calculated based on the size of the nearest service centre and its average estimated road distance from the location. In the current study, three categories of remoteness (Urban: ARIA+ 0-0.02; Regional: ARIA+ 0.03-5.92, and; Remote: ARIA+ >5.92) were used to graphically explore how the association of other independent variables with psychological distress differed by remoteness.

### Data analysis

Analyses were conducted using SPSS 19 and graphs were produced using Microsoft Excel 2010. Chi squared tests were used for between group comparisons of categorical variables and one-way ANOVAs were used for continuous variables. Binary logistic regression was used to identify significant predictors of high distress, with effect sizes reported as adjusted odds ratios (AORs) and associated 99% confidence intervals. Results of the social support calibration procedure utilizing common three year follow-up data are initially reported. To assess whether predictors of high distress differed by participant remoteness in the combined baseline sample, product terms [remoteness (continuous) by age, gender, marital status, education and social support] were produced using standardized values. Variables were entered into the model in two steps: a six variable model examining the influence of age, gender, marital status, education, remoteness, and social support on high distress; and an eleven variable model that included the interaction terms. Male gender (1) was the reference category in contrast to female gender (0); married/defacto relationship (1) was the reference category in contrast to not being in a married/defacto relationship (0), and completion of 12 or more years of education (1) was the reference category in contrast to completion of less than 12 years of education (0). To explore significant interaction effects between independent variables (A) and remoteness (R), associations between independent variables and the probability of high psychological distress were plotted by remoteness category using the equation P(distress) = 1/1+ e^–(intercept+ βA*A + βR*R + A*R*βAR)^). An α < .01 was used as a partial control for the number of statistical tests and trends p < .05 are reported.

## Results

### Preliminary analyses: Social support scale calibration using data from the HCS three year follow-up

Of the 2031 participants in the HCS preliminary three year follow-up dataset, 96.7% provided K10 scores and were included in the social support scale calibration analyses. These participants had a mean age of 69.67 (SD = 7.26) years, 48.2% were male, 73.5% were married or in a defacto relationship, 79.5% had completed 12 or more years of education, and they had an overall mean psychological distress score of 13.71 (SD = 4.83). Almost 8% of participants reported a high level of psychological distress. Highly distressed participants were less likely to be married (63.2 vs. 74.4%; *Χ*^2^(1) = 9.07, p = .004) and were less likely to have 12+ years of education (71.5 vs. 80.2%; *Χ*^2^(1) = 6.47, p = .015) than participants experiencing low-moderate levels of distress.

#### Network support

Network support items administered to the HCS and ARMHS cohorts at baseline and jointly administered to the HCS three year follow-up are presented in Table 1. Total scores for network support items were calculated to give equal weight to similar questions within each scale: HCS Network total = ZSum(items 1 to 4); ARMHS Network total = ZSum(items 1-3) + (mean(item 4)*4). These standardized total scores displayed a significant positive correlation in the HCS follow-up dataset, r(1819) = .61, p < .001, providing evidence that they assess reasonably comparable aspects of network support.

**Table 1:** Network and Personal support indices administered by the ARMHS and HCS at baseline but common to three year follow-up

**Baseline ARMHS Measures**			**Baseline HCS measures**		
**Network support**					
1	How many close friends do you have? (People that you feel at ease with, can talk to about private matters, and can call on for help) ^[scored 0-4]^	None	1	How many persons who live within one hour travelling time from your home do you feel you can depend on or feel very close to (other than members of your own family)? ^[scored 1-3]^	None
		1 or 2			1 - 2 people
		3 to 5			More than 2 people
		6 to 9			
		10 +			
2	How many relatives do you have that you feel close to? ^[scored 0-4]^	None	2	(Other than at work) How many times during the past week did you spend some time with someone who does not live with you? For example, you went to see them or they came to visit you, or you went out together? ^[scored 1-3]^	None
		1 or 2			Once\twice
		3 to 5			Three\four\five\ six\seven+
		6 to 9			
		10 +			
3	How many of these friends and relatives do you see at least once a month? ^[scored 0-4]^	None	3	(Other than at work) How many times did you talk to someone - friends, relatives or others - on the telephone in the past week (either they called you, or you called them)? ^[scored 1-3]^	None\once
		1 or 2			Twice\three\ four\ five
		3 to 5			
		6 to 9			Six seven+
		10 +			
4	Do you belong to any of these kinds of social groups? a) a social or recreational group; b) a labour union, commercial group, professional organisation; c) a church group; d) a group concerned with children (e.g. boy scouts, patents and friends etc); e) a charity concerned with community betterment, charity, or service; f) any other group. ^[scored 0-6]^	+1 for each group	4	(Other than at work) About how often did you go to meetings of social clubs, religious meetings, or other groups that you belong to in the past week? ^[scored 1-3]^	None\once
					Twice\three\ four\ five
					Six seven +
**Personal support**					
5	If something unpleasant or irritating happens and you get upset or angry about it, do you have someone you can go to who isn’t involved and tell them just how you feel? ^[scored 0-1]^	No	5	Does it seem that your family and friends (that is, people who are important to you) understand you? ^[scored 1-3]^	Hardly ever
		Yes			Some of the time
					Most of the time
6	Is there anyone who lives in or near the district you now live in who knows you very well as a person? (this includes friends as well as family members) ^[scored 0-1]^	No	6	Do you feel useful to your family and friends (that is, people who are important to you)? ^[scored 1-3]^	Hardly ever
		Yes			Some of the time
					Most of the time
			7	Do you know what is going on with your family and friends? ^[scored 1-3]^	Hardly ever
7	Is there any particular person you feel you can lean on? ^[scored 0-1]^	No			Some of the time
		Yes			Most of the time
8	Do you feel there is one particular person who feels very close to you? ^[scored 0-1]^	No	8	When you are talking with your family and friends, do you feel you are being listened to? ^[scored 1-3]^	Hardly ever
		Yes			Some of the time
9	When you are happy, is there any particular person you can share it with, someone whom you feel sure will feel happy simply because you are? ^[scored 0-1]^	No			Most of the time
		Yes	9	Do you feel you have a definite role (place) in your family and among your friends? ^[scored 1-3]^	Hardly ever
					Some of the time
					Most of the time
10	At present, do you have someone you can share your most private feelings with (confide in)? ^[scored 0-1]^	No	10	Can you talk about your deepest problems with at least some of your family and friends? ^[scored 1-3]^	Hardly ever
		Yes			Some of the time
					Most of the time
			11	How satisfied are you with the kinds of relationships you have with your family and friends? ^[scored 1-3]^	Very dissatisfied
					Somewhat dissatisfied
					Satisfied

*ARMHS*: Australian Rural Mental Health Study; *HCS*: Hunter Community Study.

#### Personal support

Personal support items administered to the HCS and ARMHS cohorts at baseline and jointly administered to the HCS three year follow-up are presented in Table 1. Due to the broader scope of the personal support items administered by the HCS at baseline, a stepwise regression of HCS personal support items onto the ARMHS personal support total score was conducted using the HCS follow-up data to identify baseline HCS personal support scale items assessing similar concepts to those tapped by the baseline ARMHS personal support scale. This analysis identified five items from the HCS personal support scale that were positively predictive of ARMHS personal support total score: item 10 (β = .39, p < .001; R^2^ = .15); item 5 (β = .17, p < .001, R^2^ = .17); item 9 (β = .10, p < .001, R^2^ = .18); item 11 (β = .06, p = .007, R^2^ = .18); and item 6 (β = .06, p = .028, R^2^ = .19). These results were used to construct the personal support scores: HCS Personal total = ZSum(items 5, 6, 9, 10, 11); and ARMHS Personal total = ZSum(items 1-6). The correlation between these standardized totals displayed a moderate positive correlation in the HCS follow-up dataset, r(1813) = .41, p < .001, providing evidence that they assess reasonably comparable aspects of personal support.

#### Composite index of social support

Two composite indices of social support were constructed by taking the average of the standardized network and personal support scores as assessed by the HCS and ARMHS at baseline (e.g. ARMHS social support index = mean(ARMHS Network total, ARMHS Personal total)). The correlation between these composite indices was moderate in the HCS follow-up dataset, r(1718) = .65, p < .001, indicating 41% shared variance. A binary logistic regression examining the influence of age, gender, marital status, education, remoteness and social support on reporting of high distress was conducted using the HCS and ARMHS social support indices separately in the HCS follow-up dataset using participants who had completed all model variables and both indices of social support (N = 1716; see Table 2).

**Table 2:** Analyses of HCS three year follow-up (N = 1716): Logistic regressions examining predictors of high psychological distress, using social support indices equivalent to those from the ARMHS and HCS at baseline

	**B**	**SE**	***p***	**AOR**	**99% CI**
ARMHS equivalent index					
Age	0.00	0.01		1.00	0.96-1.03
Male	0.31	0.20		1.37	0.83-2.26
Married/defacto	-0.41	0.21		0.67	0.39-1.14
12+ yrs education	-0.45	0.22	.04	0.64	0.36-1.12
Social support	-0.52	0.10	**	0.59	0.46-0.77
(Z)Remoteness	0.03	0.09		1.03	0.81-1.30
Constant	-1.83	0.99		0.16	
HCS equivalent index					
Age	-0.01	0.01		0.99	0.96-1.03
Male	0.18	0.20		1.20	0.71-1.02
Married/defacto	-0.39	0.22		0.68	0.39-1.18
12+ yrs education	-0.26	0.23		0.77	0.43-1.40
Social support	-0.95	0.10	**	0.39	0.30-0.50
(Z)Remoteness	0.02	0.10		1.02	0.78-1.32
Constant	-1.95	1.01		0.14	

* p < .01, ** p < 001.

**Figure 1:**
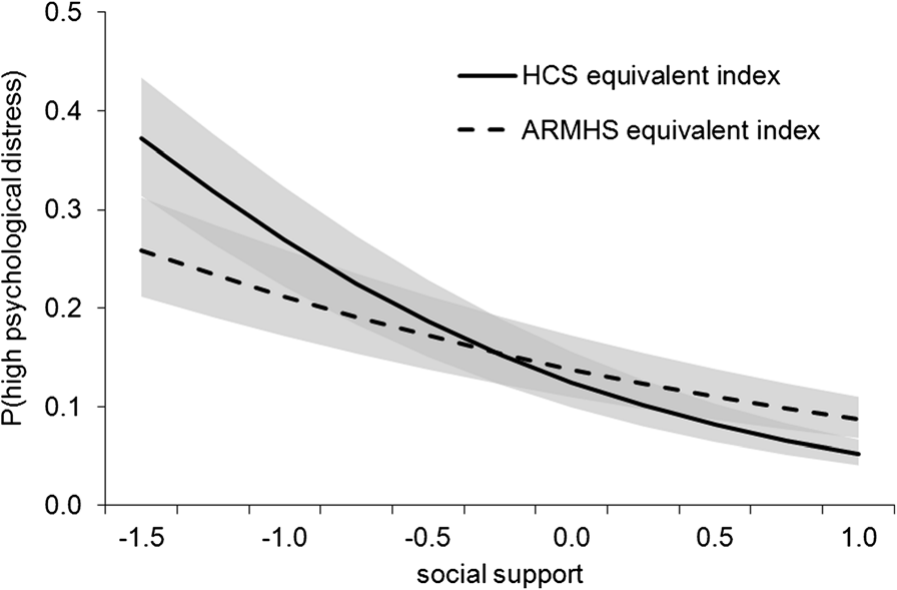
**The effect of social support (A) on the prediction of distress outcome as assessed by the HCS and ARMHS equivalent social support indices.** 99% CIs for each index are represented by grey areas with upper and lower limits determined using the equation: P(distress) = 1/1+ e^–((intercept+ βA*A)±SE^_βA_^*Z)^).

The association with high psychological distress was similar for the HCS [AOR = .39, p < .001 (99% CI .30-.50), R^2^ = .15] and ARMHS [AOR = .59, p < .001 (99% CI .46-.77), R^2^ = .05] social support indices. Overlapping 99% CIs between the HCS and ARMHS social support indices suggests the association between social support and psychological distress did not differ between the two indices. The association of each index with the likelihood of distress was plotted for social support values ranging from -1.5 to 1 SD from the mean (see Figure 1).

### Primary analyses: Influence of remoteness on predictors of baseline psychological distress

Baseline participants who provided information on all model variables (N = 4219; 89.5%) were included in analyses. These participants had a mean age of 69.00 (SD = 7.61) years, 46.1% were male, and 74.6% were married or in a defacto relationship; age and gender distributions were comparable to those for NSW [38] though persons in married or defacto relationships were somewhat over represented ([65.9% of persons aged over 55 in NSW [39]). Three-quarters (73.3%) had completed 12 or more years of education, and participants had a mean composite social support index of 0.01 (SD = 0.82) and a mean remoteness score of 1.14 (SD = 2.39). By remoteness category, 66.0% (N = 2786) of participants lived in urban areas, 27.5% (N = 1159) in regional areas and 6.5% (N = 274) in remote areas. Participants had a mean psychological distress score of 14.41 (SD = 5.30) and 9.6% of participants reported a high level of psychological distress. Highly distressed participants were less likely to be married (62.5 vs. 73.9%; *Χ*^2^(1) = 23.49, p < .001), were less likely to have 12 or more years of education (62.3 vs. 74.5%; *Χ*^2^(1) = 26.09, p < .001) and they had lower levels of social support (M = -0.76, SD = 1.05 vs. M = 0.09, SD = 0.75; F(1, 4217) = 425.01, p < .001) than participants experiencing low-moderate levels of distress, but did not differ in age, gender or remoteness.

A hierarchical logistic regression assessing predictors of high distress and whether these varied with remoteness was conducted (see Table 3). Results suggest that the six variable model including age, gender, marital status, education, social support and remoteness was a better fit than the constant only model (*Χ*^2^(6) = 364.06, p < .001). The 11 variable model assessing whether predictors of distress varied with remoteness also significantly improved the model (step *Χ*^2^(5) = 17.46, p = .004) with significant interactions of remoteness by social support (p = .002) and of remoteness by age (p = .014) observed in the prediction of high distress. No other interactions were significant. The final model accounted for 18.4% of the variance in high psychological distress (*Χ*^2^(11) = 381.52, p < .001). When holding other variables constant, this model suggests that: being in a married or defacto relationship decreased the odds of high distress by 31%; having 12 or more years of education decreased the odds of high distress by 48%; and each one standardised unit increment in social support decreased the odds of high distress by 64%. The interaction of age and remoteness indicates that as remoteness increases, older persons are less likely to be highly distressed. The interaction of social support and remoteness indicates that as remoteness increases, persons with low levels of social support are less likely to be highly distressed.

**Table 3:** Logistic coefficients for predictors of high distress (N = 4219)

**Step**	**Entered**	**B**	**SE**	***p***	**AOR**	**99% CI**
1	(Z)Age	-0.05	0.06		0.95	0.82-1.11
	Male	-0.13	0.11		0.88	0.65-1.18
	Married/defacto	-0.37	0.12	*	0.69	0.51-0.94
	12+ yrs education	-0.66	0.12	**	0.52	0.38-0.71
	Social support	-1.01	0.06	**	0.36	0.31-0.42
	(Z)Remoteness	-0.08	0.06		0.92	0.79-1.08
	Constant	-1.77	0.13	**	0.17	.
2	(Z)Age x (Z)Remoteness	-0.18	0.01	*	0.84	0.67-1.00
	Gender x (Z)Remoteness	0.13	0.12		1.14	0.84-1.54
	Marital status x (Z)Remoteness	-0.08	0.13		0.92	0.67-1.27
	Education x (Z)Remoteness	0.25	0.13		1.28	0.92-1.78
	Social support x (Z)Remoteness	0.20	0.06	*	1.22	1.04-1.44
	Constant	-1.77	0.13	**	0.17	.

* p < .01, ** p < 001.

**Figure 2:**
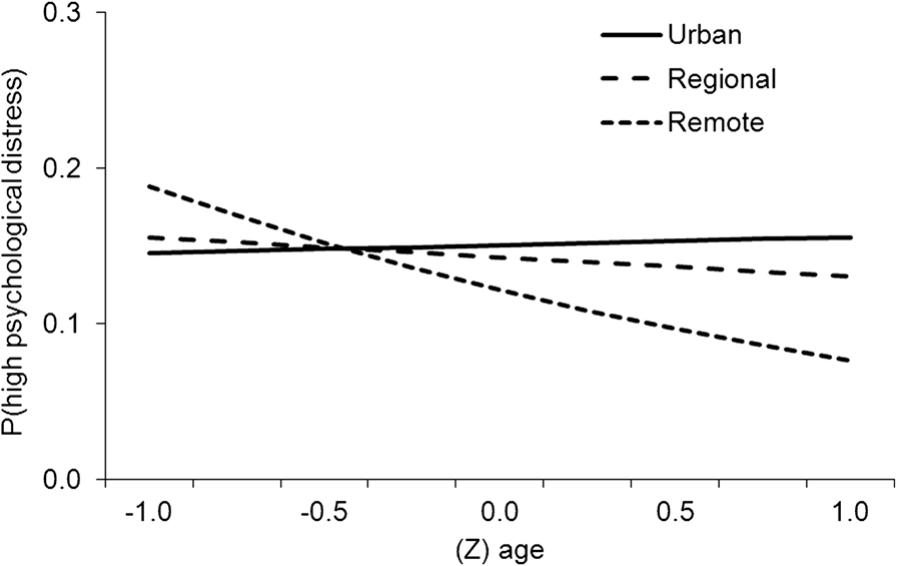
The effect of age on the prediction of distress outcomes by remoteness category.

To explore the interaction of age and remoteness in the prediction of high psychological distress, Figure 2 was constructed to plot the association between age and high distress for each remoteness category using coefficients from Table 3. The median ARIA+ value was used in these analyses to quantify each level of remoteness: Urban = 0.00 (Z = -.50); Regional = 1.96 (Z = 0.29) and; Remote = 7.72 (Z = 2.60). Age values were plotted from -1 to 1 SD around the mean score. Figure 2 indicates that there was little influence of age on distress in urban areas and the greatest influence in remote areas, with regional areas displaying an intermediate association.

**Figure 3:**
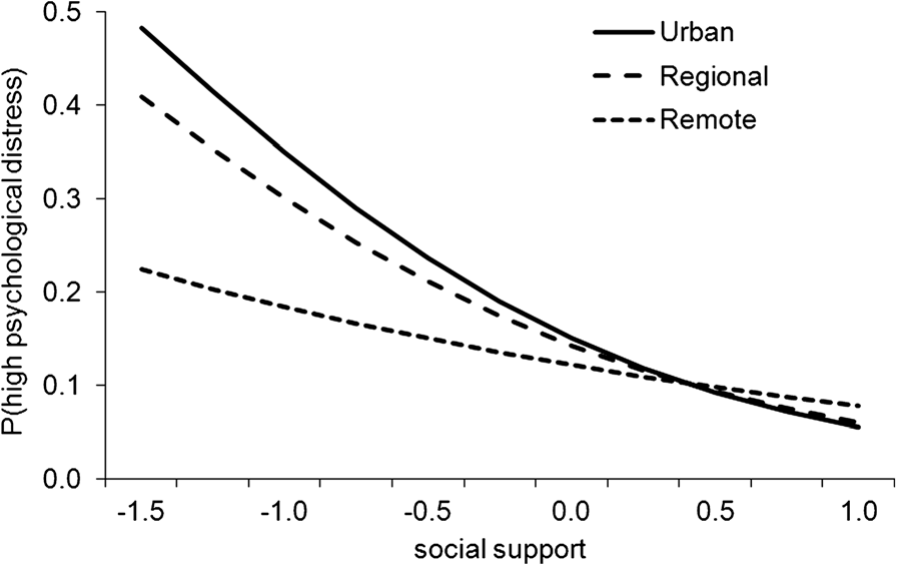
The effect of social support on the prediction of distress outcomes by remoteness category.

To explore the interaction of social support and remoteness in the prediction of high psychological distress, Figure 3 was constructed to plot the association between social support and high distress for each remoteness category using coefficients from Table 3. As in Figure 2, median ARIA+ values were used to quantify the level of remoteness. Social support values were plotted from -1.5 to 1 SD around the mean score. Figure 3 indicates there is a negative influence of low social support on distress outcomes in urban and regional areas, however this association was weaker in remote areas.

## Discussion

The current study examined whether individual level characteristics influence psychological distress outcomes differentially across urban-remote regions of Australia in a community sample of persons aged 55 and over. We hypothesised that the association of individual level characteristics with high psychological distress would be moderated by indices of area remoteness. Results provide support for our hypothesis and suggest that remoteness may have a moderating effect on the association of both social support and age with high psychological distress. Persons with low levels of social support were less likely to be highly distressed as remoteness increased; an effect particularly evident in remote, compared to urban and regional, participants. Further, older persons were less likely to be highly distressed as remoteness increased, with urban participants showing little change in psychological distress with increasing age. This study is the first to examine how determinants of psychological distress vary across to urban very remote regions of Australia.

The current results confirm often observed findings that increased likelihood of high psychological distress is associated with lower levels of education and with not being in a married or defacto relationship. Results also indicate that when controlling for age, education, marital status, social support and remoteness, there was no influence of gender on high distress in either the combined urban-remote baseline sample or the three year follow-up data from the urban-regional HCS. Recent Australian population data found women to have higher K10 scores across all age groups compared to men [31], though these effects are not always observed [40]. Additionally, a Canadian population study noted that when using a criterion cut-off for major depression, differences in the rate of major depression between men and women decreased with increasing age [41]. Given the older age of the current sample such an effect may have contributed to current results. While it is unclear why some studies of psychological distress do not show gender effects, we also observed no differential effect of gender by remoteness, suggesting that community remoteness was not a factor in the lack of gender effect, as previously proposed [40].

Interpretations of the lack of main effects of age and remoteness and the main effect of social support on the likelihood of high psychological distress are more difficult in the presence of their significant interactions. However, while the lack of association between high psychological distress and remoteness confirms observations in American [11] and Australian [10] community samples, the current research suggests that it may moderate the effects of other potential demographic and social risk factors. Previous literature has observed a positive relationship between age and psychological distress, however it is likely that the restricted age range of the current sample may explain the lack of association observed here. Indeed research suggests there is a spike in psychological distress in the adult life for persons aged in their 50s [40] and, as our study was a cohort of persons 55 and over, this restriction may explain the absence of a positive association of age and distress. The exploration of the observed interaction of age and remoteness suggested that increased age was associated with a decreased likelihood of distress in our regional and remote participants, although this had little or no impact on distress in urban areas. These results suggest that there may be some benefits associated with aging in non-metropolitan communities; however, this may also represent an urban-drift phenomenon wherein persons experiencing high levels of distress move to urban or regional areas in their older age. Indeed a Western Australian study of migration patterns of remote, regional, and urban populations found that persons in remote areas were more likely to move to urban areas following onset of disease relative to background rates of urban migration in the healthy population [42]. While the mechanisms underlying the current observation that older persons were less likely to be highly distressed as remoteness increased are unclear, the current research highlights the importance of examining contextual variations, such as remoteness, when assessing the influence of demographic factors such as age on psychological outcomes.

The current study confirmed findings that decreased levels of social support were associated with an increased likelihood of psychological distress. Exploration of the significant interaction of social support and remoteness demonstrates that the direction of this association was consistent across urban, regional and remote areas, though the strength varied. Stress and coping theories addressing the protective effects of social support on psychological wellbeing suggest that these effects may be due to ‘stress buffering’ processes wherein social support decreases the stress associated with challenging or stressful situations by increasing the individual’s coping resources thus moderating the impact of stressful life events on mental health outcomes [43]. Such theories have received limited support as literature examining an association between life stressors and levels of social support have rarely observed this buffering effect (see [44] for review). More recent 'social cognitive’ theories such as Relational Regulation Theory [45] have proposed that the protective influence of social support may actually reflect a general heightening of wellbeing and self-esteem resulting from social interactions and support and that the level of support needed to maintain this wellbeing benefit varies depending on the individual’s desire for social interaction (i.e. as shaped by social norms and individual’s personality characteristics etc.). Both researchers and theorists [2,3,46,47] have proposed that high levels of social support and social capital in rural samples underlie observations of lower rates of psychiatric morbidity compared to urban samples, however this proposal has rarely been formally tested. The current findings indeed suggest that the characteristics of the place in which we live may moderate the protective effect of social support on psychological wellbeing. However, they indicate that low levels of social support have a greater effect on wellbeing in urban and regional centres than in remote areas and, as discussed below, there are a number of scenarios which may contribute to this finding.

Firstly, this result may reflect a real difference in the association between social support and psychological distress that is borne of the values and environmental context associated with remote, in contrast to urban or regional, living. The isolation and associated social norms that come with living in remote communities may mean that the self-esteem of persons living in these environments may be less influenced by their level of social support. Alternatively, there may be more salient stressors that underlie psychological wellbeing in these communities (i.e. drought, access to resources, physical wellbeing), the effects of which are not moderated by social support.

Secondly, Relational Regulation Theory [45] suggests that different persons need different levels of social support to maintain wellbeing. As such, results may reflect a self-selection process wherein individuals who have a lesser reliance on social support for maintenance of their psychological wellbeing will move to or remain in remote areas, whereas individuals who require high levels of social support for maintenance of wellbeing will move to regional or urban areas where there is a greater opportunity to have these needs met.

Thirdly, researchers assessing the potentially negative consequences of ‘social support’ have noted that too much social interaction and participation may be detrimental to wellbeing when these interactions exceed the coping resources of the individual. In a community sample of persons aged 50 years and over, Beard et al. [48] observed that everyday contact with family and friends was related to increased depressive symptoms over time, potentially reflecting increased involvement of social networks with persons who have greater need, or increased social demand on individuals which may be beyond their coping resources. It is feasible that increased levels of social interaction in remote areas may be associated with additional burdens (i.e. stress associated with leaving farm or work commitments, longer distances to travel etc.) which may not be as keenly felt in regional or urban environments and thus the protective effect of social interactions is reduced in remote populations.

Finally, these results may indicate that social relationships described here, such as access to close confiding relationships and group participation do not describe the types of social support that are important for the maintenance of wellbeing in remote communities. There is some evidence that the influence of all facets of social support on psychological wellbeing are not uniform between urban and rural environments [18,21], perhaps reflecting the increased salience of some aspects of social support in determining psychological wellbeing in these environments. Such findings may indicate that the influence of different aspects of a person’s social sphere may differently influence, or be influenced by, psychological wellbeing depending on the environmental and social context in which that individual lives. Recent research from a South Australian study conducted as part of a broader survey by the South Australia Department of Health [24] examined a range of social capital indices, with confirmatory factor analyses producing factors representing three aspects of social capital: cognitive (‘Trust’ in the wider community; belief in the ‘Reciprocity’ of helping, and; perceived community ‘Cohesion’ in terms of character and values); bonding (the availability of ‘Help’ from close connections if needed); and bridging (‘Networks’ participation in community groups, and; individual’s participation in ‘Civic activities’ such as marches, voting, and local action groups). Structural equation models of demographic and social capital influences on mental health urban and non-urban populations revealed that ‘Trust’, ‘Help’ and ‘Cohesion’ were associated with good mental health in both the urban and rural models, while ‘Networks’ were only associated with mental health in the urban model, perhaps suggesting social networks are either less important for mental health in rural areas, or are less prone to the effects of mental health. Current evidence highlights the necessity of examining the relative influence of different aspects of social capital on psychological wellbeing outcomes in different environments.

### Strengths and limitations

A strength of the current research is its capacity to compare determinants of psychological distress across a broad spectrum of urban-remote populations, which was achieved by combining studies sampling urban and non-urban environments. By uniting cohorts in this way, the Extending Treatments, Education and Networks in Depression project (xTEND) [36] is not only able to examine these baseline associations but to ensure overlap in measures for their respective three year follow-up surveys. There are a growing range of approaches for integrating and comparing data across different cohorts (e.g. [49,50]). The current study used a process of calibrating different though conceptually similar measures of social support to provide comparable assessment of their association with psychological distress outcomes across both cohorts. The availability of a common follow-up phase allowed us to employ methods to directly compare the association of these measures of social support both with each other and a common measure of psychological distress to create a single index of social support. However, while we have combined data from studies designed for different purposes, and with differing response rates, they were conducted within similar time frames, drew samples from electoral rolls using a similar methodology and had comparable socio-demographic profiles. Nevertheless, questions remain as to whether the differences in measures used influenced our current findings. In the HCS three year follow-up dataset, the correlation between the social support measures was only moderate. However, the overlapping confidence intervals for the adjusted odds ratios of our composite measures of social support in the prediction of high distress suggest that the association between social support and high distress did not differ between the two indices. These findings will need to be verified using common measures of social support (when three year follow up data are available for both the HCS and ARMHS cohorts), as well as replicated in other samples using common measures of social support both to confirm current findings and ensure generalizability to other areas of Australia. Researchers interested in examining effects of remoteness should consider collaborations with similar cohorts to improve their representativeness.

A limitation of the current research is that younger people were not represented and so current findings may not be generalizable to this section of the population. Further, traditional measures of social support, as used in the current research, do not take into account modern forms of socialisation such as instant video, chat and text messaging and social networking services, nor do they consider the importance of persons outside the community for sustaining mental health. With the increasing accessibility and use of these services, it is likely that these modes of social communication will become increasingly important for the maintenance of psychological wellbeing, perhaps particularly in isolated communities. Future research is needed to develop tools to assess the use of and support derived from these sources.

Finally, future research should consider the influence of previous environmental exposure on relationships between remoteness and psychological wellbeing. Kim et al [18] found that the influence of remoteness on the association between social support and depression outcomes was moderated by the individual’s migration history. While the ARMHS collected information on how many years the individuals had lived in their current district, no information on previous area of residence or residential history was collected from HCS participants. As such, the current research is unable to determine what effect, if any, previous environmental exposure had on the current associations. Future research is needed to examine what effects such migration patterns have on the current findings.

## Conclusions and recommendations

This research extends our work investigating determinants of mental health [9] across the spectrum of urban to very remote communities of Australia by combining existing cohort datasets. Current findings confirm certain demographic and social factors as protective against high levels of psychological distress in a sample drawn from across the spectrum of urban-rural environs of NSW, Australia. Individual’s marital and educational status both contributed to prediction of high psychological distress. The current research suggests that increasing age may be negatively associated with high distress in regional and remote areas. Further, social support may have a greater influence on psychological distress in urban and regional areas compared to remote areas. This latter finding suggests that initiatives aimed at improving mental health outcomes in urban and regional areas should aim to improve supportive relationships at the personal level. Initiatives in remote communities may be better targeted at improving other aspects of social wellbeing, such as community level social capital. Future research is needed to examine the psychosocial factors important for the maintenance of wellbeing in remote communities. These results suggest that the nature of the community in which one lives may moderate the protective effect of individual risk factors. Future research will examine features of the social (person, family and community factors) and environmental (availability and nature of services, climate events) factors that may be associated with mental health outcomes.

## Competing interests

The authors declare that they have no competing interests.

## Authors’ contributions

BJK and TJL led the ARMHS study and JAttia led the HCS study from 2010. BJK, KJI, JA and TJL led the program of research associated with the combination of these studies as the eXtending Treatments, Education and Networks for Depression (xTEND) project. JA and TJL undertook the statistical modelling and generated the results. All authors provided interpretation of the results. JA drafted the manuscript and all authors contributed to its editing and have read and approved the final submission.

## Pre-publication history

The pre-publication history for this paper can be accessed here:

http://www.biomedcentral.com/1471-2458/12/928/prepub
